# Effects on molecular interactions of hollow gold nanoparticles and antibody for sensitizing P24 antigen determination

**DOI:** 10.1039/d4ra05277c

**Published:** 2024-09-23

**Authors:** Tao Wang, Chuanjiang Ran, Xinyue He, Shengzhou Li, Hongguang Xiang, Yan Shen, Jue Wang, Hongxia Wei

**Affiliations:** a Department of Clinical Laboratory, Second People's Hospital of Taixing City Jiangsu Province 225400 China; b Department of Pharmaceutics, School of Pharmacy, China Pharmaceutical University Nanjing 210019 Jiangsu Province China; c National Institutes for Food and Drug Control 2 Tiantan Xili, Dongcheng District Beijing 100050 China myxwj2007@163.com +86-10-67095126; d Department of Infectious Disease, The Second Hospital of Nanjing, Nanjing University of Chinese Medicine Nanjing 210003 China wghongxia@sina.com +86-13851507368

## Abstract

In recent years, with the rapid development of point-of-care testing, the application of lateral flow immunochromatography assay (LFIA) has become increasingly widespread. The key to the success of these detection technologies is the effective binding with diagnostic materials and detection antibody proteins. Although many researchers have tried to optimize antibody binding, a universally accepted strategy that can provide maximum performance has not been determined. In this study, the HIV infection P24 antigen was selected as the detection biomarker. Then the binding mechanism between hollow gold nanoparticles as diagnostic materials and detection antibodies was explored through dynamic light scattering, Fourier transform infrared spectroscopy, circular dichroism spectroscopy, and other methods. It was found that the binding efficiency is related to the change in protein secondary conformation during binding, hydrogen bonding, and van der Waals force maintain the binding mechanism between antibodies and nanoparticles. The main forces of particle complexation and the main binding site of the antibody were discussed and analyzed. Finally, an immunochromatographic system was constructed to evaluate the significant advantages of this platform compared to the common colloidal gold immunochromatographic system.

## Abstract 2

This article constructed a hollow gold immunochromatographic strip system and studied the effect of the binding mechanism between hollow gold and antibodies on the sensitivity of the strip.

## Graphical abstract

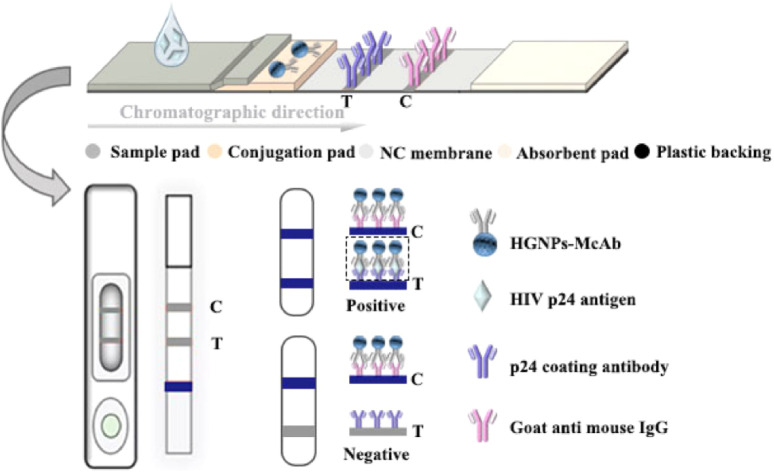

## Introduction

**Fig. 1:**
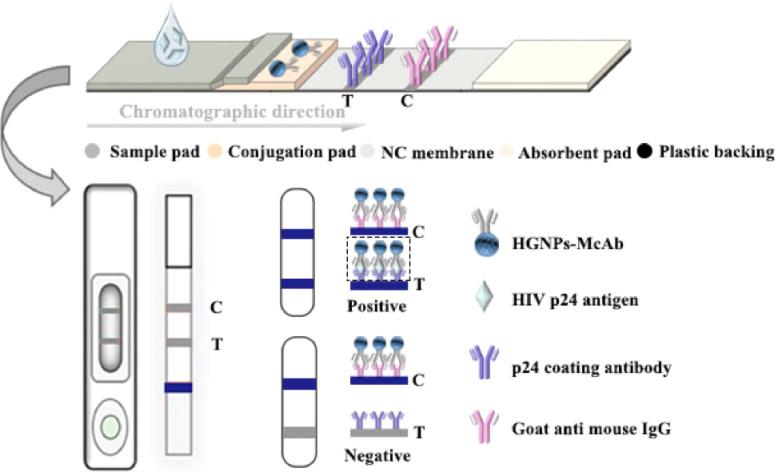
Fundamental principle of the HGNPs–LFIAs. The double antibody sandwich structure formed by HGNPs–McAb and p24 coating antibody is shown in the black dashed rectangular box.

Post-of-care testing (POCT) is crucial for providing rapid diagnostic results and timely on-site treatment.^1,2^ Over the past two decades, lateral flow immunochromatographic assay (LFIA) has received increasing attention and has become the most prominent POCT. It has been widely used in clinical diagnosis, animal and plant disease monitoring, and other fields. The results of LFIA can be easily read with the naked eye or quantified using portable devices.^3^ The immunochromatographic test strip mainly consists of a sample pad, conjugation pad, nitrocellulose (NC) membrane, absorbent pad, and plastic backing. As shown in Fig. 1, the plastic backing serves as the support body, and other parts are stacked and adhered to it in sequence, antibodies (Abs) labeled with colored nanomaterials (such as gold nanoparticles (GNPs)) are fixed on the binding pad, two prefixed immune reagent detection lines: test line (T line) and control line (C line) are marked on the NC membrane. The T line is used to determine the test results, and the C line is used to evaluate the effectiveness of the test strip. The sample to be tested moves forward from the sample pad due to chromatography. According to the different number of binding epitopes of the detected target, LFIA can be divided into two categories: the double antibody sandwich method and the competitive method.^4^ The double antibody sandwich method generally detects macromolecular targets containing multiple antigenic epitopes, such as proteins, pathogens, viruses, *etc.* The competitive method generally detects small molecule antigens (Ag) containing only a single antigenic epitope, such as agricultural and veterinary drug residues, hormones, toxins, *etc.*

LFIA is a popular, easy-to-use, and low-cost analytical method, which can be used for screening, diagnosis, and surveillance of various diseases.^5^ As we all know, domestic LFIA testing equipment plays a vital role in the surveillance of cardiovascular and infectious diseases. This method can provide information about the presence/absence or quantity of the target analyte within a few minutes after the detection starts, and the results can be easily read with the naked eye or quantified using a portable device. Now it can accurately detect a range of antigens, such as hormones, vitamins, enzymes, viruses, microorganisms, biomarkers of cardiovascular disease, biomarkers of cancer, *etc.*^6,7^ Gold nanomaterials are the most commonly used nano-labeling materials in LFIA because of their simple synthesis process, controllable morphology, good particle dispersion, good optical properties, easy surface functionalization, and high biocompatibility.^8^ However, the sensitivity of the colloid gold immunochromatographic assay (GICA) is limited by using the 20–40 nm gold nanoparticles (GNPs) with weak optical signal.^9,10^ At present, the sensitivity of GICA is mainly improved by introducing different forms of gold nanomaterials (gold nanoflowers,^11–13^ gold nanorods,^14,15^*etc.*) and gold matrix composites (platinum modified gold nanomaterials,^16^ gold magnetic nanomaterials,^17–19^*etc.*) with stronger optical signals, these new gold-based nanomaterials provide ideal properties for chemical and biological detection, such as significant body surface area ratio, strong SPR signal^20^ endowed optical properties, wide absorption of the visible region of the electromagnetic spectrum,^21^ fine tunable surface chemistry, wide structural compatibility and higher colloid stability.^22^ So far, various LFIA biosensors based on these well-designed gold nanostructures have been developed and reported with higher detection sensitivity and multiple signal output functions, such as magnetism, fluorescence,^23^ SERS,^24^ and thermal signals.^15,25^

The novel detection strategy utilizing the unique properties of GNPs brings great hope for the advancement of diagnostic detection. The key to the success of these detection techniques is that the surface of GNPs can effectively modify antibody proteins to selectively bind to specific analytes in diagnostic applications.^26^ The analytical performance of these tests depends on the loading density of immobilized antibodies and the binding mode and interaction force between GNPs and antibodies.^27^ Despite extensive efforts by researchers to optimize antibody immobilization, a universally accepted strategy that can provide maximum performance has not yet been determined. The most common methods for immobilizing antibodies onto GNPs include covalent coupling strategies. These strategies typically use chemical or heterobifunctional crosslinking agents to target the primary amine and terminal thiol of lysine residues commonly present in proteins and covalently bond with GNPs.^28^ Despite significant efforts to develop universal, powerful, and controllable covalent coupling methods, direct adsorption of antibodies on GNPs remains a commonly used method.^29^ Antibody proteins can adsorb onto the surface of GNPs to form complexes through hydrogen bonds, ionic bonds, hydrophobic bonds, and gold–sulfur bonds. In the study of conformational changes, bonding types, mediated forces, and reaction kinetics at the molecular level after the binding of antibodies and GNPs, a single analytical method cannot accurately obtain sufficient information on the interaction between gold antibody complexes.^30^ Therefore, multiple techniques need to be used in combination for analysis and discussion.^31,32^

Therefore, this article creatively proposed using HGNPs with stronger extinction performance and a simple and stable synthesis process as immune labeling materials, selecting human immunodeficiency virus (HIV) P24 antigen as a biomarker and preparation of hollow gold nanocomposite particles bound to P24 monoclonal antibody (McAb) (HGNPs–McAb) to construct the LFIA system (HGNPs–LFIA), and significantly improving compared to common colloidal gold immunochromatographic products on the market. To investigate the possible reasons for sensitization, we explored the interaction between antibodies and biomarkers on the improvement of label binding and immune chromatographic performance. The use of fluorescence spectroscopy, circular dichroism spectroscopy, and other methods can provide comprehensive information on the stability of GNP antibody binding, which hopes to guide significance for further improving the sensitivity and stability of LFIA.^33,34^

## Materials and methods

### Materials

Sodium citrate dihydrate, sodium borohydride, and hydrochloric acid were purchased from Nanjing Chemical Reagent Co., Ltd. Cobalt chloride hexahydrate was purchased from McLean. Chloroauric acid trihydrate was purchased from Shanghai Bade. Hydroxylamine hydrochloride was purchased from MERDA. Polypyrrolidone K30 was purchased from Anhui Shanhe Accessories Co., Ltd. Anhydrous potassium carbonate was purchased from National Pharmaceutical Group Chemical Reagent Co., Ltd. Sucrose and sodium chloride were purchased from Xilong Science. Trehalose was purchased from Shanghai Yuanye Biotechnology Co., Ltd. Proclin300 was purchased from SIGMA-ALDRICH. Tween-20 was purchased from Ron Reagent. Bovine serum albumin was purchased from Shanghai Aladdin reagent. 1× PBS (pH = 7.4) was purchased from Solarbio. 1 M Tris–HCl (pH = 6.8), protein Marker, and Coomassie brilliant blue dye were purchased from Beyotime. p24 monoclonal antibodies (C4-016, C4-014) and negative serum were obtained from Hunan Shengxiang Biological Co., Ltd. HIV P24 recombinant antigen (AZ13), goat anti-mouse IgG (GM106) were purchased from Hangzhou Qitai Biotechnology Co., Ltd. Bottom plate, conjugation pad, cellulose acetate film and other test strip consumables were purchased from Shanghai Jieyi Biotechnology Co., Ltd.

Preclinical samples were obtained by the Second Hospital of Nanjing with the approval of its institutional review committee, and all sample sources were informed and agreed upon by patients(approval number: 2024-LS-ky-045).

### Preparation of PVP@HGNPs

HGNPs were prepared by the cobalt template method based on the stoichiometric relationship as presented in formulas (2-1) and (2-2). Formula (2-1) provides a generally accepted reaction mechanism of cobalt boride products.

2-1 2CoCl_2_ + 4NaBH_4_ + 9H_2_O → Co_2_B + 4NaCl + 12.5H_2_ + 3B(OH)_3_

2-2 3Co + 2AuCl_4_^−^ = 2Au + 3Co^2+^ + 8Cl^−^

The glassware and magnetic mixer used for synthesis need to soak the inner wall for 8–10 min in advance with aqua regia. The synthesis method of HGNPs and the screening of various process parameters have been based on previous laboratory studies: add deionized water (Ultra Pure Water Machine, MasterTouch-S15UVF), 0.05 M sodium citrate solution, 0.4 M cobalt chloride solution, and 0.13 M sodium borohydride to round bottom flask. Then immediately connect the oil pump (Vacuum oil pump, VP-2) to remove the oxygen, continue stirring and observe the reaction, start the time after the color of the solution begins to change, continue to stir for 6–8 min, close the oil pump when the bubbles in the container basically disappear, quickly add 25 mM HAuCl_4_ solution, remove the glue plug and shake violently, until the color of solution changes and remain stable, continue to stir 30 min, and then add PVP K30 solution with 20% (w/v) to continue stirring for 2 h. The above solution was centrifuged at 10 000 rpm (high-speed freezing centrifuge, KDC140HR), and the precipitation was redispersed to 2.8 mL with deionized water.

### Preparation of the McAb labeled HGNPs

The preparation process of HGNPs–McAb is as follows: PVP@HGNPs solution was diluted to 262.5 pM, and pH is adjusted with K_2_CO_3_ or HCl. Then P24 monoclonal antibody (McAb, C4-016) is added for electrostatic adsorption for a period of time, 100 μL 10% BSA is added and sealed for 20 min, and then centrifuged. The supernatant was removed and 50 μL resolute solution was added. Finally, the prepared HGNPs–McAb was stored at 4 °C.

### Assembly of hollow gold immunochromatographic test strips

#### Cutting and pretreatment of materials

As shown in the figure, the test strip mainly consists of a sample pad, bonding pad, NC film, water absorbing pad, and bottom plate. Firstly, the purchased sample pad, bonding pad, NC film, and water absorbing pad materials are cut into suitable sizes of 1.7 cm × 30 cm, 0.5 cm × 30 cm, 2.5 cm × 30 cm, and 1.7 cm × 30 cm using a cutting machine according to the specifications of the bottom plate (6 cm × 30 cm). Wet the pre-cut bonding pad completely with bonding pad treatment solution, dry it in an oven at 40 °C for 4 hours, and seal the dry area for future use.

#### Scratch the membrane

Stick the processed sample pad, NC film, and absorbent pad of appropriate width onto the bottom plate in sequence according to the structure shown in Fig. 1, overlapping each other by 2 mm. Afterward, a gold-labeled membrane reader was used to draw a T line and a C line on the NC membrane at a rate of 1 μL cm^−1^ using 10 mM PBS diluted 2 mg mL^−1^ of P24 coated antibody (P24 monoclonal antibody, model: C4-014) dilution and 0.5 mg mL^−1^ of goat anti-mouse IgG dilution, respectively. Then, use a cutting machine to cut the assembled board into 4 mm wide test strips, dry them in an oven at 37 °C for 2 hours, and store them in a dry environment for later use.

#### Assembly

HGNPs–LFIAs assemble binding pads in different ways according to the method of adding the test sample. Dry sampling involves cutting preprocessed long strips (0.5 cm × 30 cm) into small strips of 0.5 cm × 4 mm using a cutting machine and storing them in sealed bags for future use. Add 4 μL of HGNPs–McAb dispersion prepared according to the method in 2 to each small binding pad, and dry it in a 40 °C oven for 30 minutes. After removal, place the small binding pad on the sample pad and NC film at both ends according to the structure shown in Fig. 1, and complete the assembly of a single test strip; The combination pad assembly method of the insertion method is consistent with the dry method; wet sampling is the process of assembling pre-processed long strips and pads with sample pads and absorbent pads in step 3, then scratching the film and cutting them before use.

## Results and discussion

### Characterization of PVP@HGNPs

**Fig. 2:**
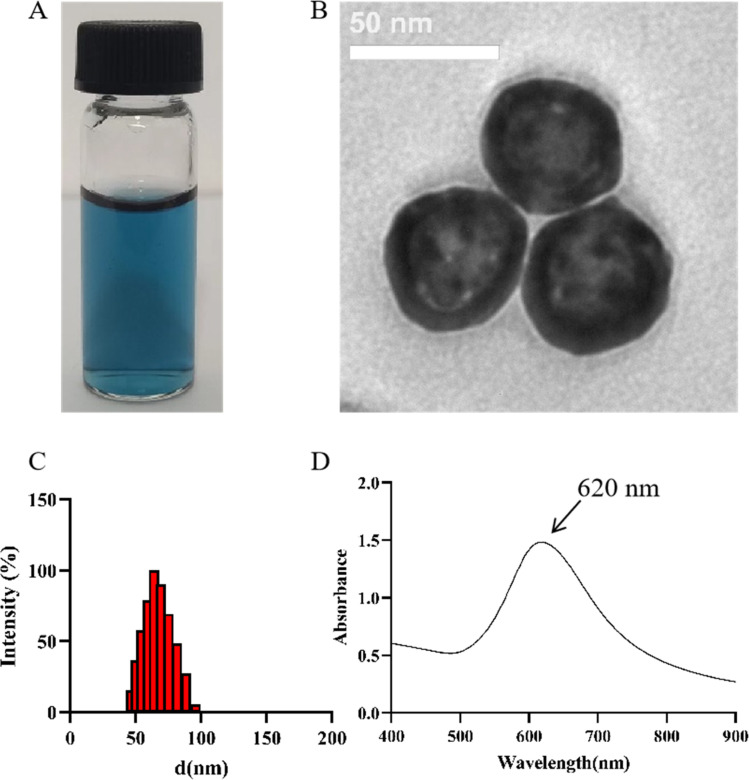
Characterization of PVP@HGNPs. Appearance (A) and TEM image (B) of PVP@HGNPs. (Scale bar = 50 nm); (C) the diameter of PVP@HGNPs; (D) the absorbance spectra of PVP@HGNPs.

As shown in Fig. 2A, the prepared PVP@HGNPs dispersion is clear and blue. The TEM image (Fig. 2B) shows that HGNPs have a good morphology and uniform particle size distribution, the particles show a hollow spherical structure with complete edges, the surface is slightly rough, and there is a layer of foggy gray hydration film around the sphere. The particle size and shell thickness of HGNPs were measured and analyzed by Photoshop (no less than 20 nanoparticles on the TEM diagram), the average thickness of hydration film is 3.08 ± 1.02 nm, and the shell thickness is 8.32 ± 1.02 nm. The particle size distribution is shown in Fig. 2C, the average particle size is 61.90 ± 1.03 nm, the polydispersion index (PDI) is 0.177 and the surface potential is −26 ± 0.25 mV, which shows that PVP@HGNPs is uniform and stable.^35^

The absorption spectrum (Fig. 2D) showed that HGNPs have a single plasma resonance peak in the range of 400–900 nm, and the maximum absorption wavelength is 620 nm. The molar extinction coefficient of HGNPs (*ε*) is calculated based on Lambert Beer's law.^36,37^*ε* (*λ*) is 1.48 × 10^10^ M^−1^ cm^−1^, which is much larger than that of GNPs with the same particle size, indicating that HGNPs have obvious advantages in optical intensity compared with ordinary GNPs to further improve the sensitivity of LFIA.

### Optimization of preparation conditions for HGNPs–McAb

**Fig. 3:**
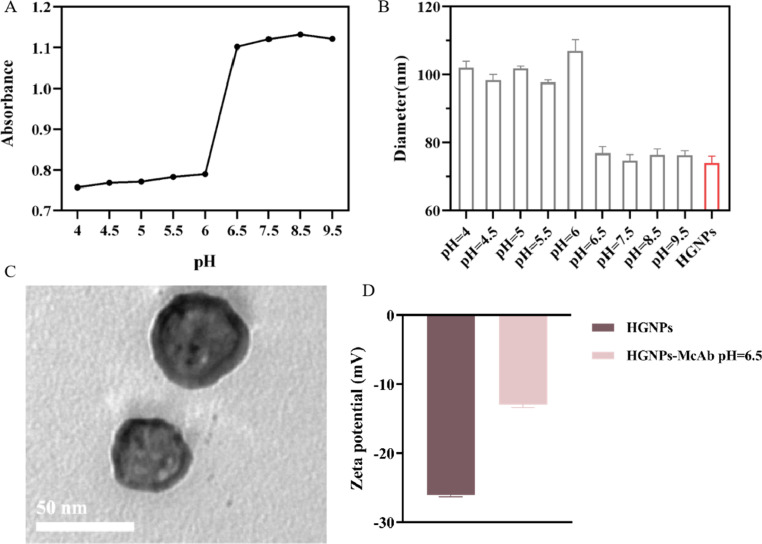
Different absorbance (A) and particle size (B) during the binding process between HGNPs and McAb under different pH conditions. (C) TEM images of HGNPs–McAb; (scale bar = 50 nm) (D) zeta potential of HGNPs and HGNPs–McAb at optimal pH. Mean ± SD, *n* = 3.

The electrostatic force plays a major role in the binding of GNPs to antibodies and is also the most easily adjustable force. At present, the common principle of binding GNPs to antibodies in LFIA is to regulate the charge carried by antibody proteins by changing the pH. At the optimal binding pH, it is generally slightly higher than the isoelectric point (PI) of proteins. The positive charge on the antibody can form a strong binding with the negative charge on the surface of GNPs while maintaining a certain repulsive force with the GNPs surface, maintaining the stability of the GNPs antibody complex structure. Moreover, since this binding is an ionic bond, it does not affect the biological characteristics of proteins. It is found that when pH < 6.5, HGNPs agglomerates in varying degrees, the absorbance decreases (Fig. 3A), and the particle size increases obviously (Fig. 3B), which indicates that HGNPs and McAb cannot form a stable composite structure when pH < 6.5. At the same time, it can be seen that when pH ≥ 6.5, the binding process is stable and no coalescence occurs.

### Characterization of HGNPs–McAb

It is found by the TEM diagram (Fig. 3C) that HGNPs–McAb have a hollow spherical structure, and the morphology does not change compared with HGNPs. The surface of HGNPs–McAb particles is coated with a thin white film, which is obviously different from the TEM diagram of HGNPs (Fig. 2B). It can be seen from Fig. 3D that the zeta potential of HGNPs–McAb is −13 ± 0.4 mV, while the potential of HGNPs is −26.1 ± 0.25 mV. The above results show that McAb is successfully loaded on the surface of HGNPs.

### Interaction studies of HGNPs with McAb

#### Fluorescence spectroscopy of the HGNPs–McAb complex

**Fig. 4:**
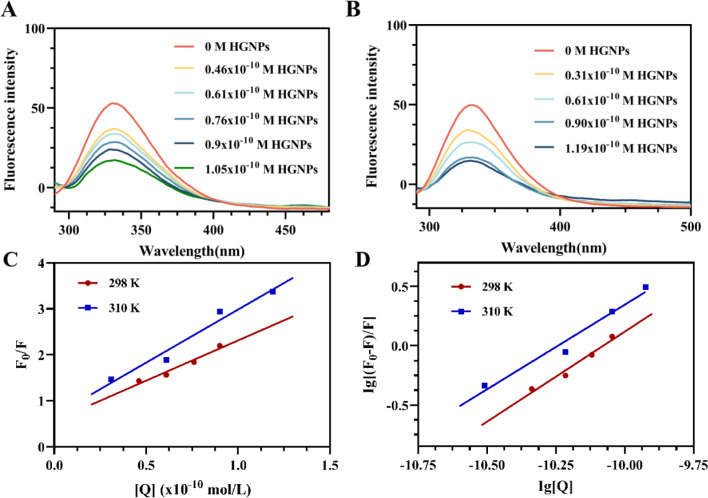
Effect of HGNPs on the fluorescence spectrum of McAb at 298 K (A) and 310 K (B); (C) the Stern–Volmer fitting of HGNPs effect on McAb fluorescence intensity at 298 K and 310 K; (D) double-logarithm plot for the quenching of McAb protein by HGNPs at different temperatures.

Fluorescence is an effective method to study the interaction between proteins and nanoparticles. Through this method, information on the binding mechanism, binding constant, binding position, binding mode, and so on can be obtained.^38^ From Fig. 4A, it is obvious that the fluorescence of McAb is quenched regularly with the increase of HGNP concentration, and there is no obvious spectral shift. A similar type of fluorescence quenching was observed at 310 K (Fig. 4B). The results showed that HGNPs interacted with McAb protein.^39^

Since fluorescence quenching is temperature dependent, the mechanism of fluorescence quenching can be determined by the change of fluorescence quenching constant at different temperatures.^38^ The fluorescence quenching data of McAb by HGNPs at 298 K and 310 K are calculated by the famous Stern–Volmer formula^40^ (formula (1-1)):1-1
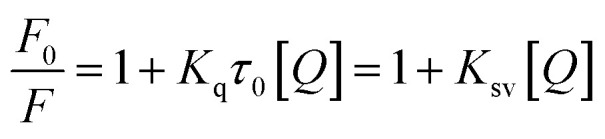
where *F*_0_ and *F* denote the fluorescence intensity at the maximum emission wavelength of McAb in the absence of HGNPs and the presence of HGNPs, *K*_q_ (M × (1s−1)) is the bimolecular quenching rate constant, *τ*_0_ is the fluorescence lifetime without quenching agent, *K*_sv_ is the Stern–Volmer quenching constant, and [*Q*] is the concentration of HGNPs.^41–43^

*K*_sv_ and *K*_q_ can be obtained by linear fitting (Fig. 4C) of [*Q*] with *F*_0_/*F*. It can be seen that the quenching constant *K*_sv_ increases slightly with the increase of temperature, but it is observed that the *K*_q_ values at different temperatures are much larger than the maximum scattering collision quenching constant (2 × 10^10^ L mol^−1^ S^−1^) (Table 1) between different quenchers and biopolymers. Therefore, from the *K*_sv_ and *K*_q_ values obtained from the Stern–Volmer diagram, it is inferred that the interaction process between McAb and HGNPs is static quenching, indicating that a relatively stable complex structure is formed between HGNPs and McAb molecules, which is of great significance to the stability of LFIA properties.

**Table 1:** Stern–Volmer quenching constants of the HGNPs–McAb system at different temperatures

*T* (K)	Equation	*R* ^2^	*k*_sv_ (L mol^−1^)	*k*_q_ (L mol^−1^ s^−1^)
298	*F*_0_/*F* = 1.748 × 10^10^[*Q*] + 0.5647	0.9552	1.748 × 10^10^	1.748 × 10^18^
310	*F*_0_/*F* = 2.308 × 10^10^[*Q*] + 0.6761	0.9651	2.308 × 10^10^	2.308 × 10^18^

When small nanoparticles bind to a macromolecule independently, the binding constant (*K*_a_) and the number of binding sites (*n*) between HGNPs and McAb can be determined by the Hill equation (formula (1-2)):^44^1-2
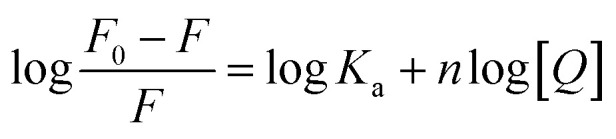


*F*_0_ and *F* represent the fluorescence intensity at the maximum emission wavelength of McAb in the absence of HGNPs and the presence of HGNPs, respectively. *K*_a_ is the apparent binding constant, *n* is the number of binding sites, and [*Q*] is the concentration of HGNPs. The linear fitting of log[*Q*] by log[(*F*_0_–*F*)/*F*] was used to obtain the double logarithm graph (Fig. 4D) of HGNPs quenching to McAb, in which the slope was the number of binding sites and the *y*-axis intercept was log *K*_a_.

Table 2 calculated the *K*_a_ value and *n* at 298 K and 310 K. It can be seen that when the temperature increases from 298 K to 310 K, the *K*_a_ value decreases from 2.0893 × 10^15^ L mol^−1^ to 4.2658 × 10^14^ L mol^−1^, and the binding constant decreases with the increase of temperature, indicating that HGNPs–McAb is unstable at high temperature. At the same time, it can be seen that there is a strong binding between HGNPs and McAb. In addition, the calculated result of the *n* value is close to 1, which reflects that there is only one major binding site between HGNPs and McAb.

**Table 2:** Binding constant and binding sites for interaction between HGNPs and McAb at 298 K and 310 K

*T* (K)	Equation	*K*_a_ (L mol^−1^)	*n*	*R* ^2^
298	log[(*F*_0_–*F*)/*F*] = 1.520 log[Q] + 15.32	2.0893 × 10^15^	1.520	0.9661
310	log[(*F*_0_–*F*)/*F*] = 1.429 log[Q] + 14.63	4.2658 × 10^14^	1.429	0.9687

#### Determination of the acting force between HGNPs and McAb

Electrostatic force, hydrophobic and hydrophilic force, hydrogen bond and van der Waals force are the main forces between biological macromolecules and inorganic small molecules. The thermodynamic process is related to the formation of the complex. The type of interaction force can be determined by evaluating the free energy change (Δ*G*), entropy change (Δ*S*) and enthalpy change (Δ*H*) in the binding reaction.^44^ Therefore, in order to study the forces between HGNPs–McAb, van der Waals and Gibbs equations are used to calculate the thermodynamic parameters^45^ (formulas (1-3) and (1-4)):1-3
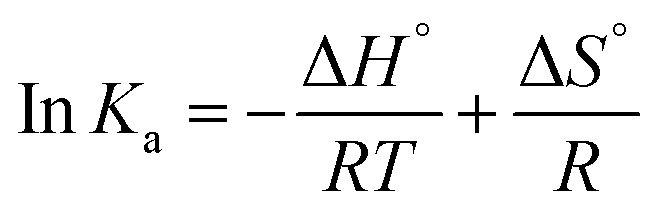

1-4 Δ*G*° = Δ*H*° − *T*Δ*S*°

In the formula, *K* represents the binding constant at different temperatures, *T* represents the absolute temperature (K), and *R* represents the gas constant (8.314 J mol^−1^ K^−1^). The calculated thermodynamic parameters Δ*S*, Δ*H* and Δ*G* are listed in Table 3. Δ*G* < 0 shows that the combination of HGNPs and McAb is a spontaneous process. Based on Subramanian and Ross theory,^46^ Δ*S* < 0 and Δ*H* < 0, it is proved that hydrogen bonding and van der Waals forces are the key factors to determine the stability of HGNPs–McAb complex. Given the existence of electrostatic gravity between McAb and HGNPs, we reasonably speculate that after the electrostatic force is used as the initial driving force to attract and combine each other, the stability of the complex structure is maintained mainly by hydrogen bond and van der Waals force.^47^

**Table 3:** Thermodynamics parameters for interaction between HGNPs and McAb protein at 298 K and 310 K

*T* (K)	Δ*G* (kJ mol^−1^)	Δ*H* (kJ mol^−1^)	Δ*S* (J mol^−1^ K^−1^)
298	−87.40	−101.69	−47.95
310	−86.82		

#### Conformational studies using FT-IR and CD spectroscopy

**Fig. 5:**
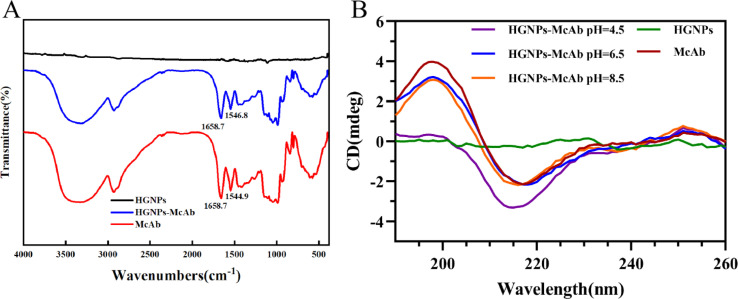
Conformational studies of McAb. (A) Infrared scanning spectra of HGNPs, McAb and HGNPs–McAb (pH = 6.5); (B) CD spectra of interaction between HGNPs and McAb under different pH conditions.

The locations of amide I band (1700–1600 cm^−1^) and amide II band (1600–1500 cm^−1^) provide detailed information on the secondary structure of proteins and can be used as indicators of protein conformational changes.^48,49^ According to Fig. 5A, the signals of amide I and II bands of HGNPs–McAb (1658.7 cm^−1^ and 1546.8 cm^−1^) are similar to those of McAb (1658.7 cm^−1^ and 1544.9 cm^−1^), indicating that McAb has been successfully connected to HGNPs.^50^ Secondly, the absorption peaks of HGNPs–McAb and McAb are basically the same, indicating that there is no change of chemical bond between HGNPs and McAb, and no covalent bond such as Au–S bond is formed in HGNPs–McAb structure, which is mainly non-covalent binding.

By measuring the CD spectrum of antibody protein, the internal relationship between the conformational change of McAb under different pH conditions and its adsorption on the HGNPs surface can be analyzed.^49^ The far ultraviolet CD spectra of HGNPs–McAb in the 190–260 nm range under different pH are shown in Fig. 5B. McAb molecules have a wide negative peak at 217 nm, which is the characteristic CD spectrum of proteins with high β-folding structure. The intensity of the negative peak band of HGNPs–McAb varies with different pH and the negative peak blue shifts to 2 nm during pH = 4.5. These indicate that the secondary structure of McAb changes in different directions and degrees with the addition of HGNPs.

The proportion of secondary structures in McAb and HGNPs–McAb is calculated by DichroWeb website. As shown in Table 4, when pH = 6.5, the content of α-helix and irregular crimp is slightly higher than that of McAb, while the content of β-folding and β corners is slightly lower than that of McAb. The structural change of HGNPs–McAb is less obvious than that before HGNPs, indicating that under the best binding pH, the coupling of HGNPs and McAb weakly changes the internal structure of McAb. At this time, HGNPs–McAb is the most stable and the load of McAb on the surface of HGNPs is large. When pH = 8.5, the irregular crimp increases from 38% to 39.3%, the β-fold decreases from 46% to 44.3%, the pH = 4.5 shows the irregular crimp increases from 38% to 39.9%, the β-corner increases from 10.4% to 10.8%, and the β-fold decreases from 46% to 43.5%. It is suggested that the conformational change of McAb molecules occurs to a greater extent in the presence of HGNPs under these pH conditions, which may be related to the instability of HGNPs–McAb structure and the low load of McAb molecules on the surface of HGNPs, which is the internal molecular factor leading to the decrease of LFIA stability and sensitivity.

**Table 4:** Effects of HGNPs on the secondary structure of McAb protein under different pH conditions

Complex	α-Helix (%)	β-Sheet (%)	β-Turn (%)	Random coil (%)
McAb	5.6	46	10.4	38
HGNPs–McAb pH = 4.5	5.9	43.5	10.8	39.9
HGNPs–McAb pH = 6.5	5.8	45.7	10.3	38.1
HGNPs–McAb pH = 8.5	6.0	44.3	10.4	39.3

### Evaluation of hollow gold immunochromatographic system

**Fig. 6:**
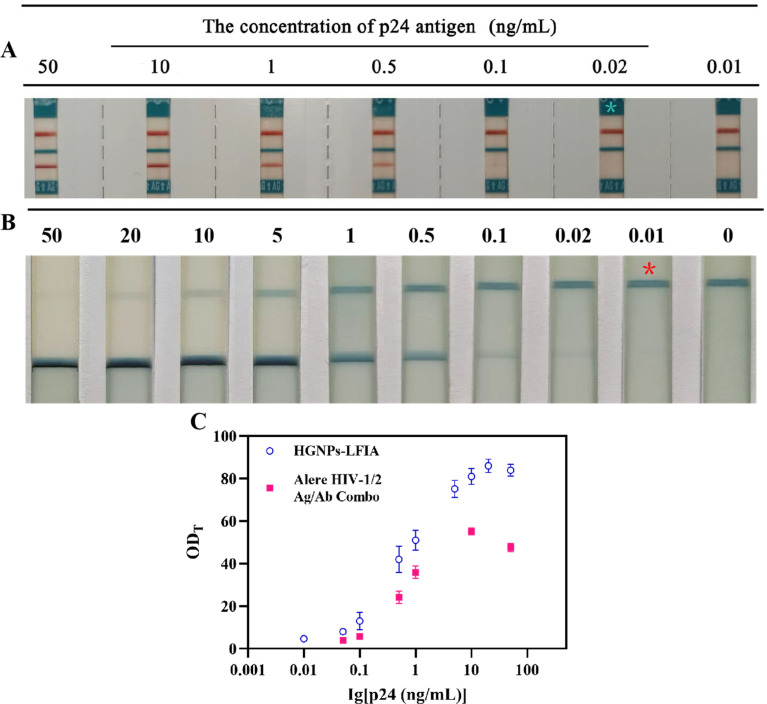
Representative images of Alere HIV Combo (A) and HGNPs–LFIA (B) for p24 antigen detection in serum. *represents the visual detection limit of the p24 antigen. (C) Comparison OD_T_ of HGNPs–LFIAs and Alere HIV combo for p24 antigen detection in serum. Mean ± SD, *n* = 3.

As shown in Fig. 6, the current main commercial rapid antigen detection test strips Alere HIV-1/2 Ag/Ab Combo have a LOD of 0.02 ng mL^−1^ for P24 antigen, while the detection sensitivity of HGNPs–LFIA is twice as high. It can be seen that the values of HGNPs–LFIAs at different P24 concentrations are significantly higher than those of Alere detection test strips, observing the depth of blue on the T-line and analyzing the optical density of the T-line (OD_T_) can be used to evaluate the excellent performance of HGNPs–McAb construction. Fig. 6C shows the OD_T_ values obtained from HGNPs–LFIA and Alere test strips as a function of different concentrations of HIV P24 antigen in serum. It can be seen that the OD_T_ values of HGNPs–LFIAs at different P24 concentrations are significantly higher than those of Alere test strips, indicating that HGNPs–LFIA has more obvious colorimetric and optical signals, which occupy more space for visual observation. HGNPs–LFIA has more advantages than the dominant fast inspection test paper.

### Hollow gold immunochromatographic system for detecting preclinical samples

**Fig. 7:**
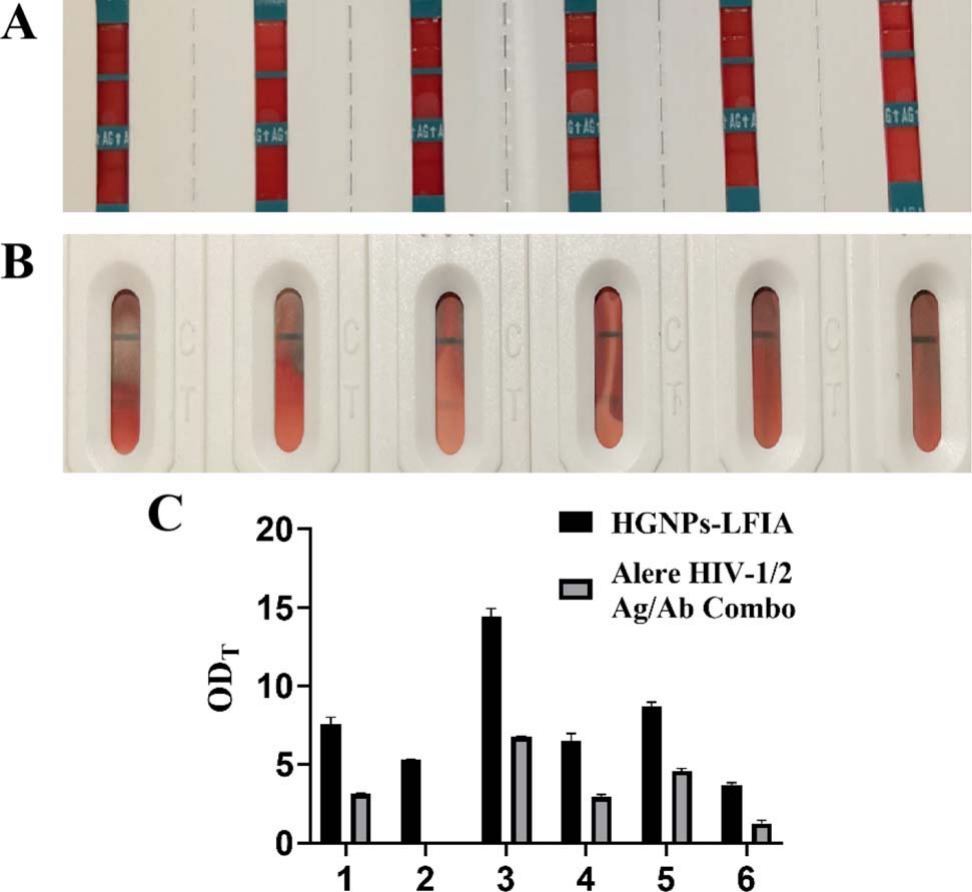
Representative images of Alere HIV Combo(A) and HGNPs–LFIA (B) for p24 antigen for preclinical samples. (C) Comparison OD_T_ of HGNPs–LFIAs and Alere HIV combo for p24 antigen detection for preclinical samples. Mean ± SD, *n* = 3.

Six samples selected from preclinical trials were used for commercial rapid antigen detection test strips Alere HIV-1/2 Ag/Ab Combo and hollow gold test strips respectively. As shown in Fig. 7, It can be seen that the line color of HGNPs–LFIA in the six samples is significantly darker than that of the commercially available test strips, and there is also one case that was not detected in the commercially available test strips. It can be seen that the OD_T_ values of HGNPs–LFIAs in the six samples are significantly higher than those of Alere test strips, indicating that HGNPs–LFIA has more obvious colorimetric and optical signals, which occupy more space for visual observation. HGNPs–LFIA has more advantages than the dominant fast inspection test paper.

## Conclusions

In this paper, the synthesis of HGNPs and the preparation of HGNPs–McAb were the main research work. Firstly, quality standards for HGNPs were established using color, concentration, absorbance, surface potential, and other main technical parameters for the subsequent preparation of HGNPs–McAb. Then, a series of spectroscopic techniques such as FT-IR, CD spectroscopy, and fluorescence spectroscopy were used to deeply study the interaction between McAb and HGNPs at the molecular level, providing theoretical support for further improving the sensitivity of HGNPs–LFIA. The results show that electrostatic adsorption is an important force in the binding process; FT-IR shows that there is no covalent bonding dominated by Au–S bonds between HGNPs and McAb; In addition, the quantitative analysis results of CD spectra indicate that the interactions under different pH conditions lead to conformational changes in McAb in different directions and degrees. The fluorescence spectrum data shows that the binding process between HGNPs and McAb molecules is static quenching, where hydrogen bonding and van der Waals forces are the main forces maintaining the stability of HGNPs–McAb complexes. We reasonably speculate that the slight conformational changes after the binding of McAb to HGNPs can enhance the strength of the interaction between HGNPs–McAb and p24 antigen, and enhance the affinity of HGNPs–McAb; Simultaneously, electrostatic adsorption, hydrogen bonding, and van der Waals forces jointly maintain the stability of the HGNPs–McAb structure; These intrinsic molecular factors can enhance the detection stability and sensitivity of LFIA. These research results provide a certain theoretical basis for the application of HGNPs–McAb in LFIA, but a more in-depth mechanism of action remains to be studied. The later research work was based on HGNPs–McAb to construct the HGNPs–LFIA system. The results showed that the detection sensitivity of HGNPs–LFIA was about 2 times higher than that of the Alere test paper, and it had a stronger color and OD signal than the Alere test paper. The visual observation of qualitative results was more advantageous, which hope can achieve the detection of HIV acute infection period.

## Data availability

The data supporting this research result can be found in the article or obtained from the corresponding author.

## Author contributions

Conceptualization, Hongguang Xiang and Jue Wang; data curation, Tao Wang and Chuanjiang Ran; formal analysis, Tao Wang, Chuanjiang Ran, Xinyue He and Hongguang Xiang; investigation, Tao Wang, Chuanjiang Ran, Xinyue He and Hongguang Xiang; methodology, Xinyue He, Shengzhou Li and Jue Wang; resources, Xinyue He; supervision, Shengzhou Li and Jue Wang; validation, Tao Wang, Chuanjiang Ran and Xinyue He; writing – original draft, Tao Wang, Chuanjiang Ran, Xinyue He and Yan Shen; writing – review & editing, Jue Wang and Hongxia Wei. All authors have read and agreed to the published version of the manuscript.

## Conflicts of interest

The authors declare no conflict of interest.
